# The clinical efficacy of periodontally accelerated osteogenic orthodontics in patients with bone fenestration and dehiscence: a retrospective study

**DOI:** 10.1186/s13005-022-00344-z

**Published:** 2022-12-08

**Authors:** Ziling Chen, Huan Zhou, Kaili Zhang, Xu Wang, Liangqiuyue Zhong, Yuxia Hou, Yue Chen

**Affiliations:** 1 Key Laboratory of Shaanxi Province for Craniofacial Precision Medicine Research, College of Stomatology, Xi’an Jiaotong University, Xi’an, China; 2 Department of Periodontology, College of Stomatology, Xi’an Jiaotong University, Xi’an, People’s Republic of China; 3 Department of Orthodontics, College of Stomatology, Xi’an Jiaotong University, Xi’an, People’s Republic of China

**Keywords:** Periodontally accelerated osteogenic orthodontics, Bone fenestration and dehiscence, Periodontal tissues, Guided bone regeneration, Gingival recession

## Abstract

**Purpose:**

The objective of the study was to explore the effect of periodontally accelerated osteogenic orthodontics (PAOO) in orthodontic patients with bone dehiscence and fenestration in the anterior alveolar region of the mandible.

**Methods:**

A retrospective study was performed in 42 patients with bone dehiscence and fenestrations in the anterior alveolar region of the mandible who underwent the PAOO technique. The bleeding index (BI), probing depth (PD), keratinized gingiva width (KGW), gingival recession level (GRL), and gingival phenotype were recorded and assessed at baseline and 6 and 12 months postoperatively. Cone-beam computerized tomography was used to measure bone volume in terms of root length (RL), horizontal bone thickness at different levels, and vertical bone height at baseline and 6 months and 12 months after surgery.

**Results:**

The sample was composed of 42 patients (22 males and 20 females; mean age, aged 25.6 ± 4.8 years) with 81 teeth showing dehiscence/fenestrations and 36 sites presenting gingival recessions. There was no significant difference in BI, PD, or KGW (between baseline and 6 or 12 months postoperatively) based on the clinical evaluations (*P* > 0.05). Gingival recession sites demonstrated a significant reduction in the GRL after surgery (*P* < 0.05). Furthermore, the proportion of teeth with a thick gingival phenotype increased from 33.61% at baseline to 53.13% at the end of the follow-up. In addition, the bone thickness measurements at the mid-root and crestal levels were markedly increased compared with the baseline values (*P* < 0.05), although the increase in thickness at the apical level was not statistically significant (*P* > 0.05).

**Conclusions:**

Within the limitations of the study, the results show that the PAOO technique is beneficial to periodontal conditions in terms of soft and hard tissue augmentation. The PAOO procedure may represent a safe and efficient treatment for orthodontic patients with bone dehiscence and fenestration.

**Trial registration:**

This study was approved by the ethics committee of the stomatological hospital affiliated with Xi'an Jiaotong University (xjkqll [2019] No. 016) and registered in the Chinese Clinical Trial Registry (ChiCTR2100053092).

## Background

At present, the considerable number of adult orthodontic patients is driving demand for aesthetics-centred, function-oriented and fast-paced treatment, which poses a great challenge in clinical practice [1–3]. Generally, age is not a contraindication to orthodontic treatment; however, the tissue response to orthodontic forces in terms of cell activity and collagen fibre conversion is markedly slower in adults than in younger patients (teenagers and children). Additionally, hyalinized zones form readily on the pressure side of the orthodontically moved tooth, potentially hindering the tooth from moving in the intended direction [4, 5]. The average duration of orthodontic treatment for adults is 18.7 to 31 months [6]. According to the American Board of Orthodontics (ABO) standards, the mean length of one-phase orthodontic treatment was 24.6 months, which is considerably longer than the average times for children and teenagers [7]. To some degree, the longer duration of tooth movement limits the possibility of providing a suitable treatment procedure for many adults seeking orthodontic treatment.

In this context, some alternative treatments have been proposed to overcome the above limitations of orthodontics. In particular, the introduction of periodontally accelerated osteogenic orthodontics (PAOO) has been advantageous for adult patients. PAOO distinguishes itself from traditional orthodontics by combining clinical periodontal treatment with orthodontic movement, involving selective surgical corticotomy, bone grafting, and orthodontic forces; based on the principle of the regional acceleratory phenomenon (RAP), an increase in bone metabolism and a transitory state of osteopenia allow accelerated tooth movement [8, 9]. An accumulating body of research has indicated that PAOO has advantages over traditional orthodontic methods in terms of accelerated tooth movement up to 3 to 4 times, increased scope of orthodontic therapy, abridged treatment duration, sustainable alveolar bone augmentation, increased range of tooth movement, reduced root resorption and enhanced stability of the postorthodontic mandibular irregularity index for at least 10 years [8, 10–12]. To our knowledge, although the research to date has indicated that PAOO is a safe, time-consuming and effective treatment, most of the studies were case reports, [13–15] and there is a lack of systematic studies to ascertain whether PAOO is safe or detrimental to the periodontal tissues of adults, especially in patients with bone dehiscence and fenestration.

Bone dehiscence (a defect that extends to the cervical surface of the root, leading to marginal alveolar bone loss) and fenestration (a window that affects the root surface but is still bordered by bone along its coronal aspect) are the most common alveolar bone defects [16, 17]. A previous study noted that Class II and Class III subjects showed a high prevalence of bone defects surrounding the anterior mandibular teeth, with rates of up to 41.11% and 45.02%, respectively [18]. These defects usually lead to root exposure, gingival recession and even treatment relapse or failure, which pose challenges in orthodontic treatment [19, 20]. Therefore, shortening the duration of orthodontic treatment and decreasing severe sequelae are of great significance for orthodontic patients, especially adults with bone fenestration and dehiscence, which are also aesthetically significant issues that are time consuming to treat. PAOO has been regarded as a promising therapeutic strategy with minimal side effects in terms of root resorption and bone defect risks [16].

Therefore, the present study was designed to examine the clinical efficacy of PAOO in adult patients with bone fenestration and dehiscence. Periodontal status was evaluated by the bleeding index (BI), probing depth (PD), keratinized gingiva width (KGW), gingival recession level (GRL) and gingival phenotype at baseline and 3, 6 and 12 months postoperatively. Bone volume was also measured during the follow-up period, with the aim of providing guidance for the clinical application of PAOO therapy.

## Methods

### Study design

**Fig. 1:**
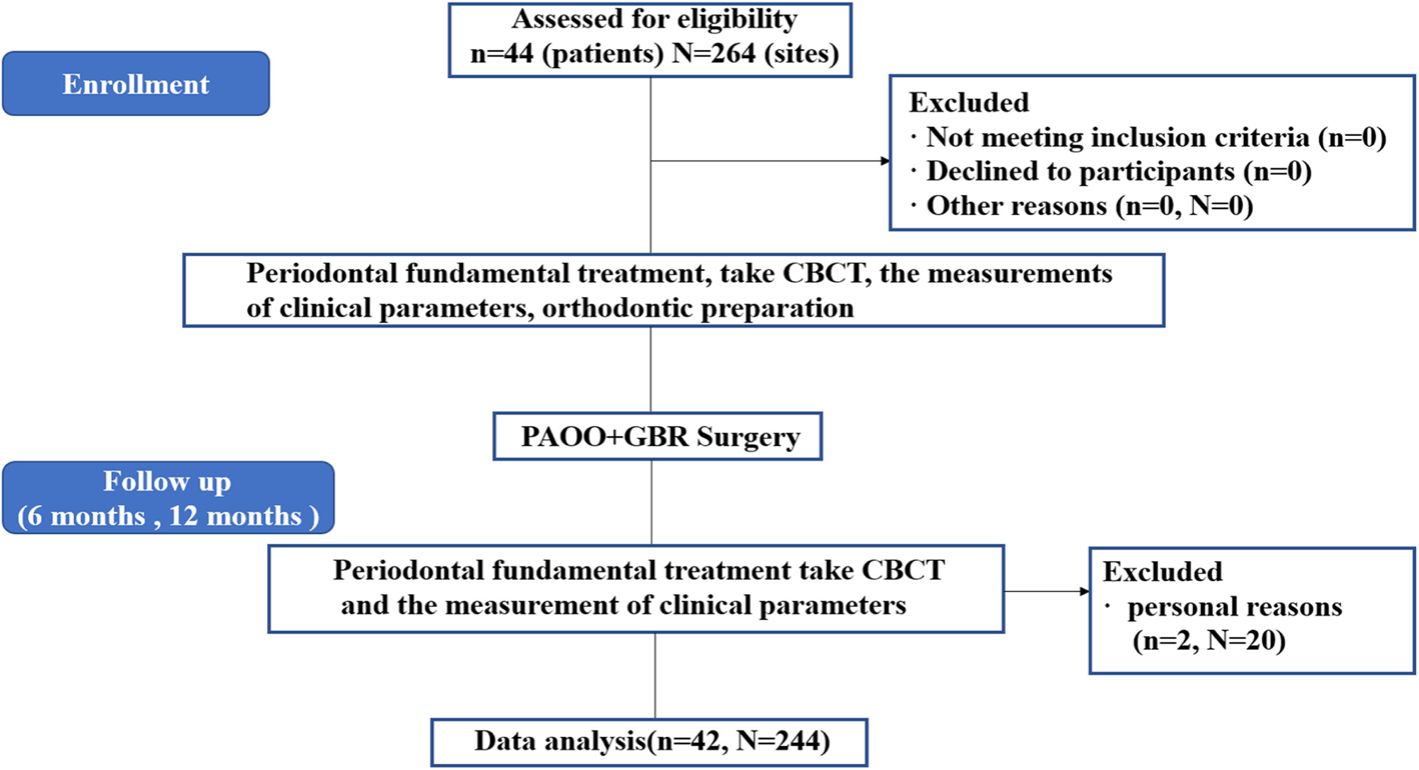
Flowchart

The present single-centre, retrospective study included 54 recruited subjects who were prescribed PAOO surgery by the Department of Periodontology, School of Stomatology, Xi'an Jiaotong University, China, from December 2017 to June 2019. The inclusion criteria called for (a) patients aged at least 18 years; (b) patients with alveolar bone fenestration or dehiscence on the labial surface of the mandible before surgery; (c) non-smokers; and (d) patients with no uncontrolled systemic diseases such as infectious or metabolic diseases, hypertension, diabetes, cardiovascular disease and immunodeficiency. The exclusion criteria were as follows: (a) patients who had undergone bisphosphonate therapy, chemotherapy, radiotherapy, or anticoagulant therapy or reported a history of head/neck radiation that could affect bone metabolism; (b) patients who were taking medications associated with the occurrence and development of drug-induced gingival enlargement, e.g., calcium channel antagonists; (c) females who were lactating or pregnant; and (d) patients who had received previous orthognathic or orthodontic treatment [21]. The current study was conducted in accordance with the Declaration of Helsinki, and the protocol was reviewed and approved by the ethics committee of the School of Stomatology, Xi'an Jiaotong University (approval number: xjkqll [2019] No. 016). In addition, the study was registered in the Chinese Clinical Trial Registry (ChiCTR2100053092). All the recruited subjects were explicitly informed of the intent and duration of the study, and informed consent was received from all the patients. A flowchart of the recruited subjects is presented in Fig. 1. During the follow-up period, two subjects were excluded from the study: one female subject was unable to perform CT due to pregnancy, and one subject could not be contacted because of phone number changes.

### Surgical and procedures

#### Treatment protocol

In accordance with the clinical treatment guidelines, each subject was treated with a pre-adjusted fixed mandibular appliance (0.022″ × 0.028″ in size, Integra Brackets, Rocky Mountain Orthodontics Inc., Denver, CO, USA) during the week preceding PAOO surgery; however, the appliance was not activated pre-operatively. Orthodontic tooth movement was started 2 weeks after the periodontal surgical procedure. During orthodontic treatment, nickel-titanium arch wires (0.012″, 0.014″, 0.016″, and 0.018″, to align and level the postoperative arch) and stainless steel arch wires (0.019″ × 0.025″, to complete the treatment) were used in accordance with routine orthodontic adjustment guidelines [22, 23].

#### PAOO surgery

All subjects underwent PAOO surgery under local anaesthesia; specifically, a full thickness mucoperiosteal flap was raised with a #15 blade at the interdental papillae on the buccal aspect (first premolar to first premolar), and two vertical releasing incisions were positioned one tooth beyond the “bone activation” region, with the aim of fully exposing the surgical field and relieving tissue tension. A split-thickness flap (where the periosteum flap was separated from the overlying mucosal layer) was then carefully elevated 3–4 mm apically. Two periosteal flap segments in the “coronal” and “apical” regions were ultimately created. After flap reflection, corticotomy was performed. Specifically, in the inter-radicular space, vertical alveolar decortications that extended 2–3 mm below the crest of the alveolar bone were created and then connected with horizontal grooves (located 2–3 mm beyond the apices of the roots). Afterwards, deproteinized bovine bone material (Bio-Oss, Geistlich, Wolhuser, Switzerland) was applied to the prepared region with light pressure, and a collagen membrane (Geistlich) was utilized to increase the stability of the graft material. Subsequently, flap tissue was advanced coronally and positioned at the cemento-enamel junction (CEJ) level, completely covering the graft material and collagen membrane. Finally, the procedure was completed by placing individual 4–0 absorbable polyester interrupted sutures, which connected the lingual tissue, the labial flap and the membrane.

#### Postoperative management

All patients were provided with cold packs for external application to ameliorate postoperative swelling (oedema). Routine antibiotics and nonsteroidal anti-inflammatory drugs were prescribed for use for at least 3 days. Each patient was explicitly provided oral hygiene instructions, and the use of antiseptic mouth wash (0.12% chlorhexidine gluconate solution) for plaque control was recommended. The sutures were removed 1 week after the surgery. All the enrolled subjects were asked to participate in regular follow-up sessions for clinical examination and radiographic evaluation.

### Clinical outcomes

#### Clinical examination

The following clinical parameters were measured and recorded during the follow-up period. (1) PD: the distance from the gingival margin to the bottom of the gingival sulcus; (2) BI: score from 0–5 according to Mazza’s definitions; [24] (3) KGW: the distance from the gingival margin to the mucogingival junction; [25] (4) GRL: the distance from CEJ to the lowest point of the gingival margin; (5) gingival phenotype: a classification based on the visibility of the periodontal probe through the gingival margin as the labial sulcus was being probed. If the outline of the underlying periodontal probe could be seen through the gingiva, it was categorized as a thin biotype; if not, it was categorized as a thick biotype.

#### Radiographic measurements

**Fig. 2:**
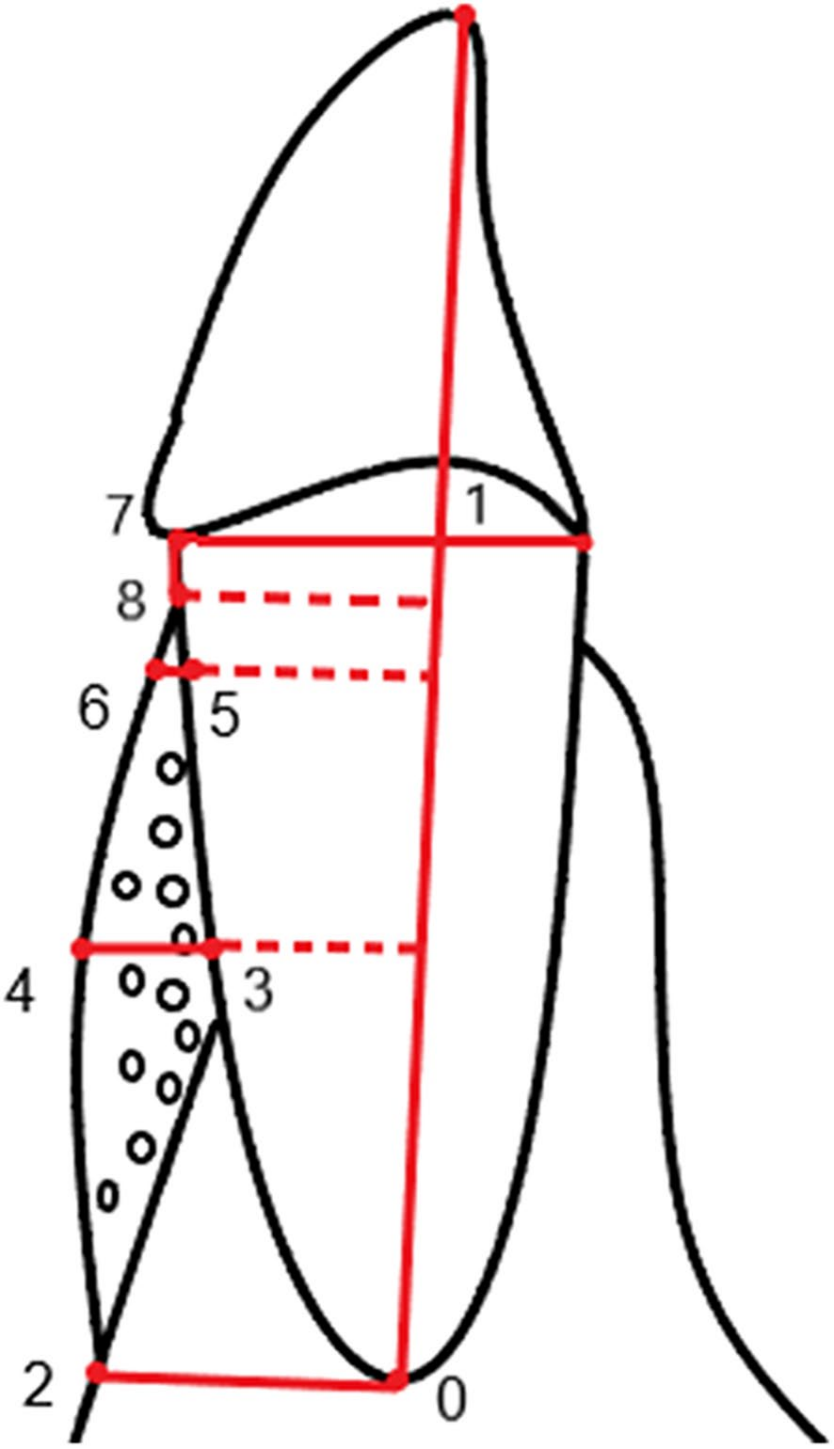
Schematic diagram of radiographic measurements. (0- the root apex; 1- the intersection of the labial-lingual CEJ line and the long axis; 2- the intersection of the labial alveolar bone surface and the line perpendicular to the long axis through dot 0; 3- the mid-root point of the labial root surface; 4- the intersection of the labial alveolar bone surface and the line perpendicular to the long axis through dot 3; 5- the point 2 mm below the labial CEJ; 6- the intersection of the labial alveolar bone surface and the line perpendicular to the long axis through dot 5; 7- the labial CEJ; 8-the alveolar crest.)

All patients were scanned using a commercially available cone-beam CT (CBCT) scanner (Vatech, Korea) before surgery and 6 and 12 months postoperatively. CBCT images were reconstituted using the image-processing software Mimics 18.0 (Materialise, Belgium). The following radiographic parameters were measured (Fig. 2) according to Xiao Xu et al. [26]:

(1)Root length (RL): the distance from the root apex (dot 0) to the intersection (dot 1) of the labial-lingual CEJ line and the long axis. (2) Bone thickness at the apical level (ABT): a line perpendicular to the long axis was made through the root apex (dot 0). ABT was the distance from dot 0 to the intersection (dot 2) of the labial alveolar bone surface and the line described above. (3) Bone thickness at the mid-root level (MBT): a line perpendicular to the long axis was made through the mid-root point of the labial root surface (dot 3). MBT was the distance from dot 3 to the intersection (dot 4) of the labial alveolar bone surface and the line described above. (4) Bone thickness at the crestal level (CBT): a line perpendicular to the long axis was made through the point 2 mm below the labial CEJ (dot 5). CBT was the distance from dot 5 to the intersection (dot 6) of the labial alveolar bone surface and the line described above. (5) Vertical bone height (VBH): the shorter the distance from the labial CEJ (dot 7) to the alveolar crest (dot 8), the greater the VBH.

### Statistical analysis

The Statistical Package for the Social Sciences (SPSS, version 18.0, Chicago, IL) was used for statistical analysis. All the descriptive data are presented as the mean ± SD. The data were tested to determine whether they fulfilled the assumptions of normality and homogeneity of variance. We found that some of the datasets followed non-normal distributions. One-way analysis of variance (ANOVA) and the Kruskal–Wallis test were used to assess the efficacy of treatment by comparing measurements from before surgery (baseline) and 6 months and 12 months after surgery. The data analysis was performed with SPSS (Version 25.0, Chicago, IL, USA), and a P value < 0.05 was considered significant.

## Results

### Sample description

**Fig. 3:**
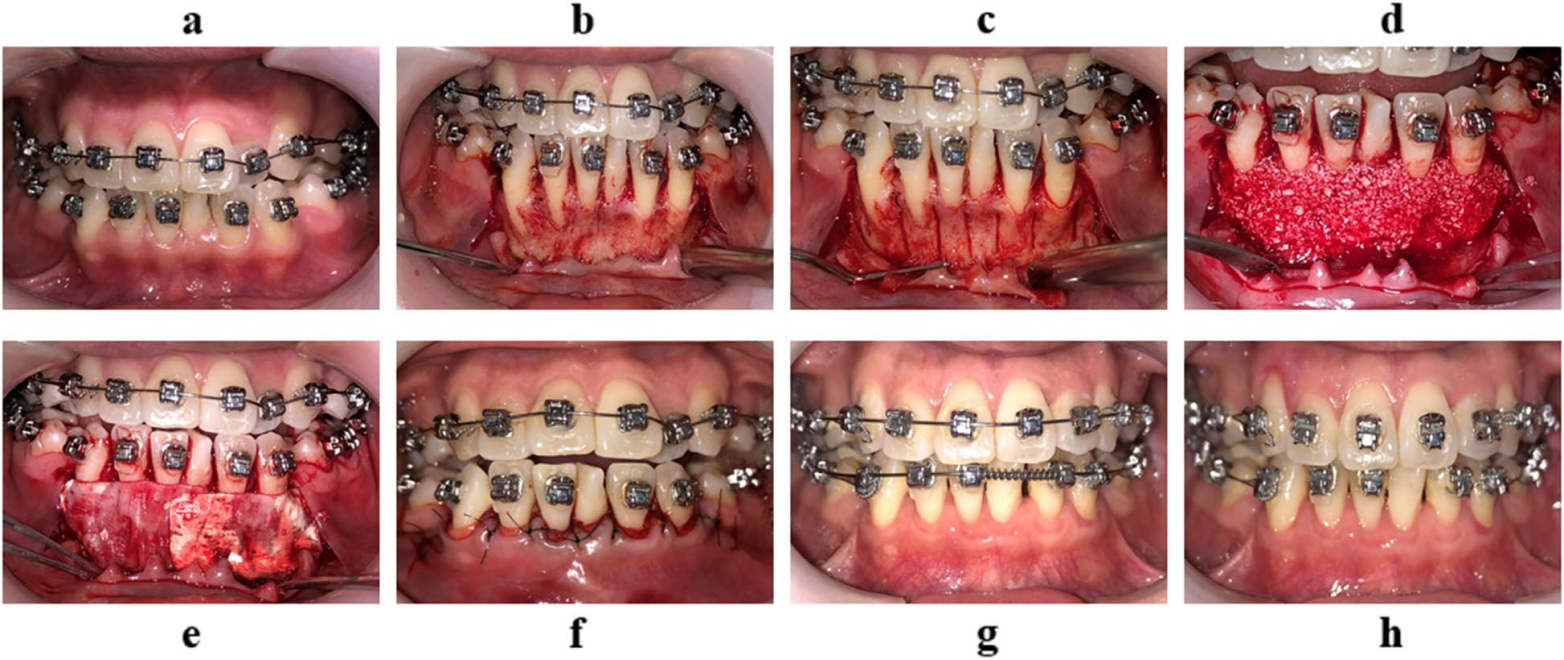
Surgical procedure of PAOO. (a) Presurgical treatment. (b) Full-thickness flap reflection (yellow dotted lines show bone dehiscence). (c) Performing corticotomies in the inter-radicular space. (d) Placement of grafting materials on the surface of alveolar bone. (e) Collagen membrane covering the grafting materials. (f) Interrupted sutures. (g) Six-month follow-up. (h) Twelve-month follow-up

A flowchart of the recruited subjects is presented in Fig. 1. During the follow-up period, two subjects were excluded from the study: one female subject was unable to undergo CT due to pregnancy, and one subject could not be contacted because of a changed telephone number. A total of 42 subjects (22 males and 20 females, aged 25.6 ± 4.8 years) with a total of 244 affected teeth participated in the study and completed the follow-up (Table 1) (Fig. 3).

**Table 1:** Basic information on the participants

Variable	Outcome assessment
No. of patients (sites)	42 (244)
Mean age (range, years)	30.9 (20.1–43.7)
Sex ratio (male:female)	20:22
Mean follow-up (range, months)	12 (11–13)
Phenotype ratio before surgery (thin: thick)	129:115
Phenotype ratio after a year (thin: thick)	82:162

### Clinical outcomes

**Fig. 4:**
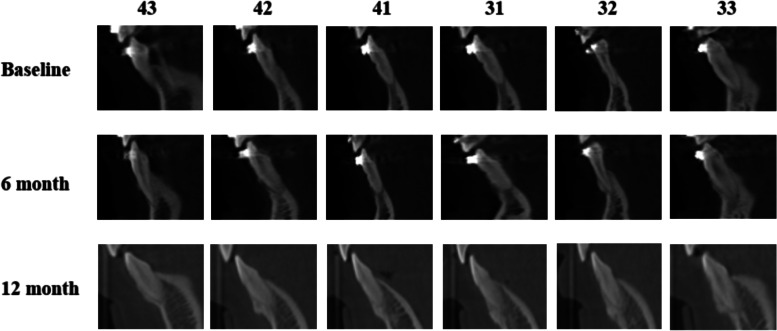
CBCT images of mandibular anterior teeth before surgery (baseline) and after surgery (6 months and 12 months)

There was no significant difference in the BI or PD of the periodontal tissue between baseline and 3, 6, or 12 months postoperatively (*P* > 0.05). At the end of the observation period, KGW was observed to increase by 0.21 mm, although the difference was not significant (*P* > 0.05). Compared with the baseline, there was a significant reduction in the mean GRL (Fig. 4) after surgery (*P* < 0.05). By the end of the observation period, the GRL had decreased by 0.61 mm (Table 2); however, this difference was not significant at any of the follow-up visits. In the present study, a total of 16 teeth had Miller class I gingival recession (the mean GRL was 1.94 mm) at baseline. GRL decreased by an average of 1.35 mm and 1.38 mm at 3 and 6 months after surgery, respectively, and these differences were statistically significant (*P* < 0.05) (Table 2). The proportion of teeth with a thick gingival phenotype increased from 47.1% (115/244) at baseline to 66.4% (162/244) at 6 months after surgery (Table 3).

**Table 2:** Descriptive statistics of clinical measurements

Multiple comparisons							
	BL	6 mo	12 mo	*P*	BL-6 mo	BL-12 mo	6 mo-12 mo
**BI**	0.68 ± 0.56	0.60 ± 0.51	0.56 ± 0.52	0.061			
**PD**	2.21 ± 0.84	2.21 ± 0.85	2.21 ± 0.84	0.827			
**KGW**	4.76 ± 1.66	5.21 ± 1.69	4.97 ± 1.76	0.420			
**GRL**	1.25 ± 1.35	0.56 ± 0.86	0.64 ± 0.86	0.000	0.000	0.000	1

Data are presented as the mean (95% CI)

12 mo = 12 months after surgery, 6 mo = 6 months after surgery, BL = baseline, PD = probing depth, KGW = keratinized gingiva width, GRL = gingival recession level

**Table 3:** Descriptive statistics of radiographic measurements

Multiple comparisons							
	BL	6 mo	12 mo	*P*	BL-6 mo	BL-12 mo	6 mo-12 mo
**RL**	12.11 ± 1.47	11.99 ± 1.46	11.84 ± 1.44	0.147			
**CBT**	0.37 ± 0.47	0.68 ± 0.52	0.66 ± 0.50	0.000	0.000	0.000	1
**MBT**	0.34 ± 0.43	1.82 ± 0.77	1.77 ± 0.76	0.000	0.000	0.000	1
**ABT**	3.73 ± 1.40	3.91 ± 1.31	3.90 ± 1.29	0.318			
**VBH**	8.36 ± 3.02	9.48 ± 2.10	9.09 ± 2.03	0.000	0.000	0.154	0.095

Data are presented as the mean (95% CI)

12 mo = 12 months after surgery, 6 mo = 6 months after surgery, BL = baseline, RL = root length, CBT = bone thickness at the crestal level, MBT = bone thickness at the mid-root level, ABT = bone thickness at the apical level, VBH = vertical bone height

### Radiographic evaluations

Based on the obtained data, there was no statistically significant change in root length at any of the follow-up points (*P* > 0.05). Compared with the baseline, the mean MBT and CBT were increased at 6 and 12 months after surgery, and these differences were statistically significant (*P* < 0.05). However, the increase in ABT was not statistically significant (*P* > 0.05).

## Discussion

Many adult patients with malocclusions are reluctant to undergo traditional orthodontic procedures due to the prolonged treatment time, which usually increases the probability of suffering from other concomitant diseases, including dental caries, decalcification, root resorption, gingival recession and other periodontal diseases. In this context, PAOO technology has been introduced to satisfy adult patients’ demand for shorter treatment times without compromising the results. Indeed, orthodontic tooth movement involves the compression of the periodontal ligament, accelerating the kinetics of crestal bone resorption and reconstruction in what is known as the “periodontal phenomenon” [27]. Thus, it is necessary and, indeed, highly significant to explore the effect of the PAOO procedure on periodontal status in adult patients, particularly in patients with bone fenestration and dehiscence, which is a periodontal hazard in itself. Based on our current study, the PAOO technique was demonstrated to be beneficial to periodontal tissues in terms of soft and hard tissue augmentation; thus, PAOO may represent a safe and efficient treatment for orthodontic patients with bone fenestration and dehiscence.

As a novel technology to shorten the treatment period without compromising orthodontic results, PAOO is heavily relied on in the comprehensive treatment of patients with occlusal and aesthetic issues. PAOO was first introduced by Wilcko in 2001 based on the RAP theory [11]. The author assumed that surgical trauma in healthy tissues could cause osteopenia, reduce bone resistance to tooth movement, and allow tooth movement to be accelerated. More importantly, the PAOO procedure was identified as an effective treatment with minimal root resorption and bone dehiscence compared with conventional orthodontic treatment.

In our current study, there were no significant differences in BI, PD or KGW (between baseline and 3, 6 and 12 months postoperatively). These findings were in line with a previous study conducted by Miyamoto T et al., in which periodontal parameters remained stable after the implementation of PAOO surgery supplemented with deproteinized bovine bone mineral with 10% collagen (DBBM-C) or without DBBM-C [28]. BI and PD are closely related to plaque biofilm and gingival inflammation; keratinization of the gingiva is of great significance because it enables periodontal tissues to resist external stimulation, and the ability to resist inflammation was identified to be positively related to KGW. All of the patients which recruited in our study are people with bone fenestration and dehiscence. According the presented study, the KGW of orthodontic patients without bone fenestration and dehiscence had significantly difference (0.48 ± 1.84 mm) after augmented corticotomy-assisted orthodontics (ACAO) compared with traditional orthodontic patients. In addition, traditional orthodontic patients even appeared new gingival recession [29]. Based on the data obtained from our present study, good control of plaque could be achieved with good oral hygiene habits, and PAOO did not increase the risk of gingival inflammation because the reduced time with a fixed appliance did not facilitate the conversion of commensal bacterial biofilms to destructive periodontopathic biofilms [30]. In addition, the proportion of teeth with a thick gingival phenotype was increased from 43.6% (at baseline) to 63.3% (12 months postoperatively) in the present study; these encouraging results may be the results of the guided bone regeneration procedure, which aims to promote periodontal bone regeneration. The gingival thickness was found to be positively correlated with the alveolar bone width. The increase in bone thickness (at the mid-root and crestal levels) observed in the present study may have given rise to the increase in the thick gingival phenotype [31]. In general, a thick gingival phenotype was correlated with a relatively good clinical therapeutic effect, which indicated a relatively promising therapeutic effect of PAOO in patients with bone fenestration and dehiscence. Gingival recession is a common complication in orthodontic treatment, and it was reported that approximately 15% of patients suffered development or aggravation of gingival recession after orthodontic treatment [32]. However, in this particular study, 112 tooth sites without gingival recession before the treatment remained free of gingival recession 12 months after the operation. In fact, a significantly reduction in GRL was recognized at the gingival recession sites at the end of the observation period. The significant reduction of gingival recession and covering of the exposed root may be correlated with the use of coronally advanced flaps. Additionally, the improved stability of the periodontium was considered to be a result of managing bone dehiscence and fenestration, which can decrease the possibility of periodontal tissue recession [8]. Collectively, these data indicated that the PAOO procedure does not increase the risk of gingival recession and tissue inflammation in patients with bone fenestration and dehiscence, and to a certain degree, applying PAOO technology may be beneficial to gingival recession sites.

In addition to the reduced periodontal concerns, the PAOO procedure was observed to facilitate an increase in bone volume. A previous study reported that the alveolar bone height and width both increased significantly after the implementation of the PAOO procedure. Additionally, a recent study conducted by Liu and colleagues demonstrated that PAOO treatment can provide adequate graft stabilization characterized by superior coronal augmentation and favourable vertical volume [33]. Coscia et al. revealed that PAOO could remarkably increase the horizontal ridge thickness (at the mid-root and apex levels) of the lower anterior teeth, while no significant change in vertical alveolar bone was identified [34]. In our current study, the bone height and width (at the mid-root and crestal levels) were increased markedly compared with the baseline values, although the increase at the apical level was not statistically significant. The observed increase in bone thickness and height could be positively related to bone grafting and the RAP [35].

According to the recent survey, VBH was partially determined by the design of surgical incisions with tension-free design, having adequate membrane coverage and the augmented grafting material displacement and leakage [36, 37]. All of the patients recruited in our study used deproteinized bovine bone material (Bio-Oss, Geistlich, Wolhuser, Switzerland) combined with the collagen membrane (Geistlich). There are two reasons for using collagen membranes: decreasing the leakage of bone materials and preventing epithelial cells from affecting osseointegration. Fibroblasts as well as bone-forming cells are able to attach to, proliferate on and migrate over collagen membranes, which would help to achieve better functional periodontal regeneration [38, 39]. Most studies have demonstrated periodontal regeneration following the combination approach. A systematic review showed histologically superior healing following the combination of barrier membranes and grafting materials when compared with barrier membranes alone or grafting materials alone [40]. Additionally, to avoid more invasive and less predictable regenerative procedures, stem cells of different origins, such as induced pluripotent stem cells (iPSCs), have been proposed as possible alternatives. IPSCs have the potential to proliferate and differentiate into all cell types derived from the three primary germ layers (ectoderm, endoderm and mesoderm), making them a potential alternative resource for the regeneration of either mineralized tooth components or supporting tissue. In addition, to avoid more invasive and less predictable regenerative procedures, Stem cells of different origins such as induced pluripotent stem cells (iPSCs) were proposed as possible alternative. IPSCs have potential for proliferation and differentiate into all derivatives of the three primary germ layers: ectoderm, endoderm and mesoderm, which could be proposed as alternative in regeneration either of mineralized tooth components or supporting tissue [41].

Root resorption, an undesirable sequela of traditional orthodontic treatment with a long treatment. Thus, the average orthodontic treatment time for adults is 18.7 to 31 months duration, is usually attributed to hyalinizing necrosis of the periodontal ligament and commonly identified in adults [6]. However, significant root resorption was not identified in the current study, which was in accordance with previous findings [28]. Based on the current understanding, after PAOO surgery is performed, cortical incision initiates the RAP to reduce the resistance to tooth movement, leading to a decrease in the orthodontic treatment time and a reduction in root resorption.

The present study, combined with previously published data show that, as a technology combines corticotomy-facilitated orthodontics, alveolar augmentation, and periodontal treatment, PAOO treatment facilitates the management of pre-existing bone fenestration and dehiscence, further improving the periodontal stability. PAOO differs from prior techniques by the additional step of alveolar bone grafting. It is this additional step that is believed to be responsible for the increased postoperative alveolar bone amount, which enhances the long-term orthodontic stability. All of the surgeries in our study were done on the buccal side. The surgery would done on the lingual/palatal side sometimes when patients undergoing lingual orthodontics or the need of lingual inclination of the anterior incisors. However, there lack of the study about lingual PAOO may due to the risk of violating important lingual anatomic structures. Nahm et.al showed that augmented corticotomy on the palate was beneficial for bodily movement in a bialveolar patient with an extremely thin alveolar bone housing [42].

The present study, combined with previously published data, shows that PAOO treatment, as a technology that combines corticotomy-facilitated orthodontics, alveolar augmentation, and periodontal treatment, improves periodontal stability by facilitating the management of pre-existing bone fenestration and dehiscence. However, although the present study demonstrated favourable results based on the outcomes obtained, there still exist some limitations, and the long-term clinical efficacy of PAOO in adult patients with bone fenestration and dehiscence remains unknown. In addition, although the quantity of new bone was ascertained, the quality of the newly formed bone also needs to be measured and analysed. In future studies, we will expand the dataset and continue the study along with histologic analysis to strengthen the basic theory and clinical basis for the proper use of PAOO.

Finally, suitable protective measures must be identified with regard to clothing, operating protocols, disinfection of environments, and management of waiting rooms and front offices under the circumstances of the COVID-19 pandemic [43].

## Conclusion

The data obtained in the present study show that PAOO may represent a promising, safe, and effective treatment for adults with bone fenestration and dehiscence; it can result in improved periodontal health and simultaneously facilitate the repair of bone dehiscence and fenestration on the labial aspect of the mandibular anterior area, which can also be beneficial to soft tissue. However, further clinical investigations should be performed over a long follow-up period to evaluate long-term stability after PAOO.

## Data Availability

All data generated or analysed during this study are included in this published article.

## Abbreviations

PAOO: Periodontally accelerated osteogenic orthodontics
RAP: Regional acceleratory phenomenon
BI: Bleeding index
PD: Probing depth
KGW: Keratinized gingiva width
GRL: Gingival recession level
CEJ: Cemento-enamel junction
RL: Root length
ABT: Bone thickness at the apical level
MBT: Bone thickness at the mid-root level
CBT: Bone thickness at the crestal level
VBH: Vertical bone height
